# Acoustic analysis in relation to kinship and familiarity for contact calls of adult female golden snub-nosed monkeys living in a multilevel society

**DOI:** 10.1093/cz/zoaf060

**Published:** 2025-08-23

**Authors:** Wanyue Wei, Dongling Zhang, Jingyuan Yang, Junyu Luo, Long Chen, Xuecong Liu

**Affiliations:** College of Life Sciences, University of Chinese Academy of Sciences, No. 19A Yuquan Road, Shijingshan District, Beijing 100049, China; College of Life Sciences, University of Chinese Academy of Sciences, No. 19A Yuquan Road, Shijingshan District, Beijing 100049, China; Shennongjia National Park Administration, No. 19 Chulin Road, Muyu Town, Shennongjia 442421, Hubei Province, China; Hubei Provincial Key Laboratory on Conservation Biology of the Shennongjia Golden Snub-nosed Monkey, No. 19 Chulin Road, Muyu Town, Shennongjia 442421, Hubei Province, China; Guangzhou National Laboratory, No. 9 XingDaoHuanBei Road, Guangzhou International Bio Island, Guangzhou 510005, Guangdong Province, China; College of Life Sciences, University of Chinese Academy of Sciences, No. 19A Yuquan Road, Shijingshan District, Beijing 100049, China; College of Life Sciences, University of Chinese Academy of Sciences, No. 19A Yuquan Road, Shijingshan District, Beijing 100049, China

**Keywords:** acoustic similarity, age proximity, individuality, kin discrimination, phenotype matching, *Rhinopithecus roxellana*

## Abstract

Kinship plays an essential role in the evolution of mammalian social behavior. The mechanisms of kin discrimination have attracted enduring interest, and phenotype matching and familiarity through prior association are the most likely ones among mammals. Previous studies on the mechanisms of kin discrimination based on acoustic signals have obtained inconsistent results. Golden snub-nosed monkeys (*Rhinopithecus roxellana*) are well-known for their multilevel social structure composed of several distinct units, the formation and stability of which largely depend on genetic relatedness among adult females. Taking advantage of the long-term demographic monitoring of a provisioned free-ranging group in Shennongjia National Park, China, this study provided a systematic acoustic analysis for contact calls of adult females in relation to kinship and familiarity. Results showed that structural similarities of contact calls between individuals did not vary among nonkin, distant kin, or close kin, while the calls exhibited clear individuality. Structural similarities of the calls between individuals significantly increased initially and then showed a tendency to decrease as the length of time co-resident in the same social unit (an index of familiarity) increased. Collectively, the acoustic structure was likely to reflect a dynamic tradeoff between maintaining social bonds and advertising individual identities. In addition, acoustic similarities decreased with age differences between individuals, likely due to age-related morphological changes in vocal apparatus. The results of this study improved the understanding of specific functions of contact calls and provided a basis for investigating kin discrimination mechanisms via acoustic signals in a primate living in multilevel societies.

## Graphical Abstract

**Graphical Abstract:**
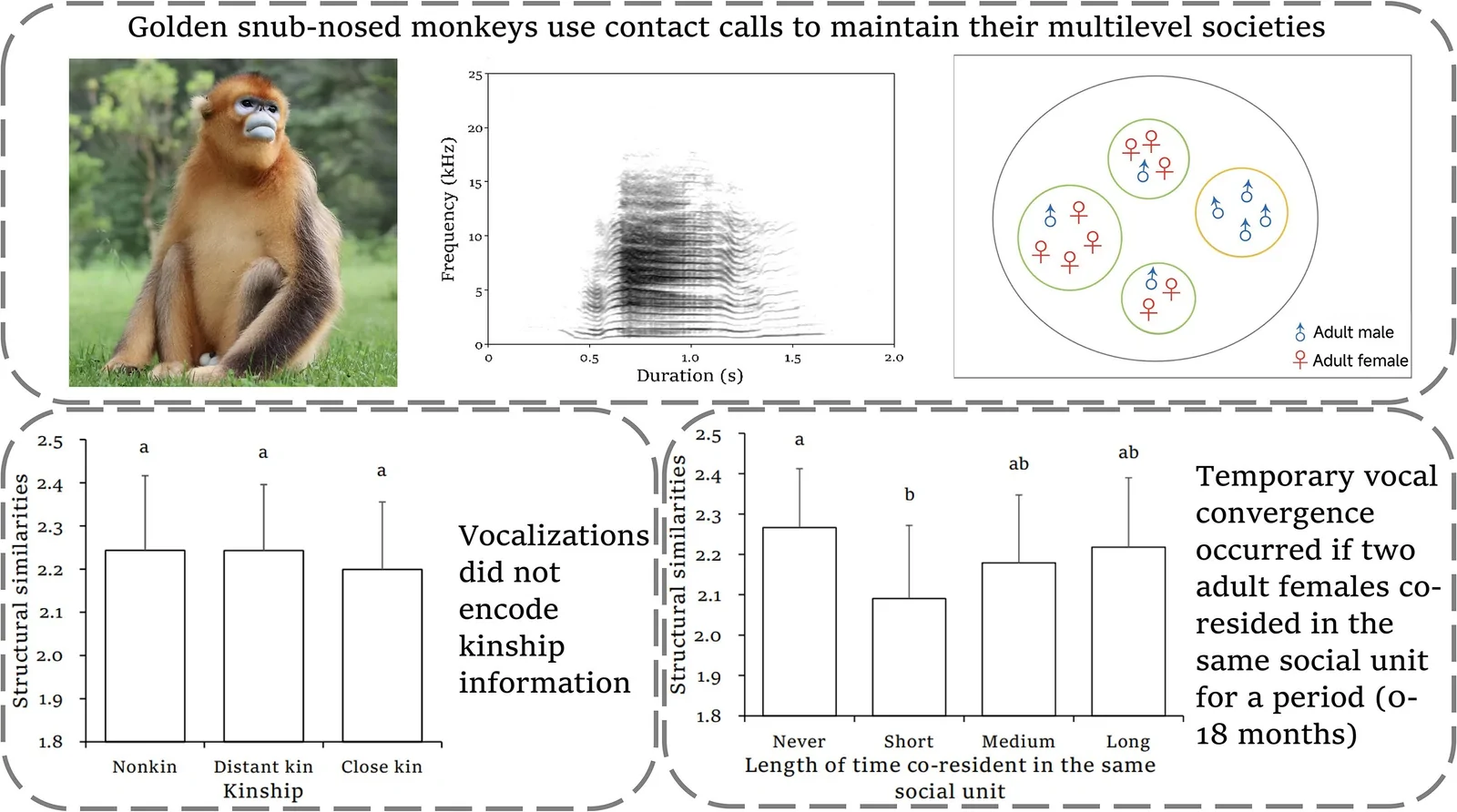

Many mammals live in social groups with a network of cooperative and competitive interactions among individuals (Sussman and Garber 2011; Fischer et al. 2017; Weiss et al. 2019; Rebout et al. 2021). Knowledge of this complex network is crucial for an individual to succeed in forming affiliative relationships with desirable partners and in competing with others for access to scarce resources or mating opportunities (Zuberbühler and Byrne 2006; Kappeler 2019). Kinship is believed to play an essential role in the evolution of social behavior. According to the theory of kin selection, individuals gain inclusive fitness benefits via preferential treatment of their relatives in social interactions such as frequent affiliation, reduced aggression, agonistic aiding, and spatial proximity (Hamilton 1964a, 1964b; Rebout et al. 2021). A precondition of kin selection is that the distinctions between kin and nonkin are sufficient so that kin and nonkin can be unambiguously identified. Since the concept of kin selection was proposed about 60 years ago (Hamilton 1964a, 1964b), the mechanisms and cues allowing animals to discriminate their kin from nonkin have attracted intense and enduring interest (Hepper 1986; Halpin 1991; Tang-Martinez 2001; Widdig 2007; Penn and Frommen 2010; Pfefferle et al. 2014; Levréro et al. 2015; Kessler et al. 2018). Most researchers agree that phenotype matching and familiarity through prior association are the most likely mechanisms of kin discrimination among mammals (Widdig 2007; Levréro et al. 2015).

Discrimination by familiarity emphasizes the necessity of a period of previous direct association. In particular, many mammals are characterized by female-biased philopatry and male-biased dispersal, and thus, animal individuals live in groups with enduring mother-offspring bonds and stable matrilines (Greenwood 1980; Pusey 1987). In such cases, there is no doubt that learned familiarity through social association can be an important mechanism underlying the discrimination of maternal kin such as aunts, cousins, grandmothers, and maternal siblings (Hammerschmidt and Fischer 1998; Charrier et al. 2003; Bohn et al. 2007). Familiarity can also be an important mechanism underlying paternal kin discrimination (Widdig 2007). In species with high reproductive skews toward one or a few dominant males, for instance, paternal half-siblings are likely to be more familiar because they tend to be born in the same age cohort and go through the same life-history stages at the same time, and interact more with each other than with non-peers (Janus 1992; Silk et al. 2006; Levréro et al. 2019). In such cases, age difference can be used as a proxy index for kin discrimination (Widdig et al. 2001; Smith et al. 2003; Widdig 2007; Levréro et al. 2019).

Phenotype matching assumes that closer genetic relatedness leads to greater phenotypic similarities, and animal individuals assess kin relationships to other individuals by comparing a phenotypic template derived from themselves or known relatives (Tang-Martinez 2001; Widdig 2007). Verifying phenotype matching as a mechanism of kin discrimination is extremely challenging, primarily because it requires ruling out or controlling for confounding effects from learned familiarity (Tang-Martinez 2001; Widdig 2007). Nevertheless, some studies have provided evidence supporting kin discrimination by phenotype matching (Heth et al. 1998; Pfefferle et al. 2013, 2014; Levréro et al. 2015). Golden hamsters (*Mesocricetus auratus*) reared in the laboratory, for example, can discriminate flank odors from kin and those from nonkin, regardless of whether they are reared with the odor donors (Heth et al. 1998). Free-ranging mandrills (*Mandrillus sphinx*) are able to discriminate among close kin, distant kin, and nonkin based on acoustic signals, after controlling for the degree of familiarity (Levréro et al. 2015).

In terms of the potential cues (visual, chemical, and acoustic) facilitating kin discrimination (Halpin 1991), acoustic signals may be relatively advantageous in several aspects: instant communication, long-distance transmission, and propagation over physical obstacles (Kondo and Watanabe 2009). The basis for acoustic signals as cues of kin discrimination is that genetically related individuals share morphological features in the vocal apparatus (e.g., the oscillating muscle tissues of vocal folds) and, in turn, acoustic features of the vocalizations produced (Fitch and Hauser 1995; Riede 2010). Although the acoustic structure of vocalizations in most nonhuman mammals is largely genetically determined, subtle structural modifications within call types, which require only little articulatory control, may emerge as a result of auditory experience or learning, at least in some species, a phenomenon known as vocal accommodation (reviewed in Ruch et al. 2018; Janik and Knörnschild 2021). The direction and extent of structural adjustments depend on specific functions of given vocalizations within a particular social framework (Ruch et al. 2018; Zürcher et al. 2021; Painter et al. 2024). For instance, structural similarities (i.e., vocal convergence) of contact calls are of importance to maintain a high level of social affiliation between individuals in Campbell's monkeys (*Cercopithecus campbelli*) living in stable groups with a strict hierarchy (Lemasson and Hausberger 2004; Lemasson et al. 2011), whereas individual signature whistles (i.e., vocal divergence) play a central role in the maintenance of multilevel social alliances in Indo-Pacific bottlenose dolphins (*Tursiops aduncus*) living in fluid fission–fusion societies (King et al. 2018).

Contact calls are widespread among social mammals and serve as affiliative signals that have evolved to function as identity advertisements and maintain contact and spacing with group members (Kondo and Watanabe 2009; Chaverri et al. 2020). In light of the functions of contact calls and the importance of genetic relatedness in mediating social behavior, these vocalizations could carry information about kin relationships (along with other information such as individual identity). There have been only a few studies on the acoustic analysis of contact calls in relation to both genetic relatedness and social familiarity between individuals in mammals, and most of them have focused on nonhuman primates (e.g., Crockford et al. 2004; Lemasson et al. 2011; De Marco et al. 2019). However, these studies have obtained inconsistent results. Structural similarities of contact calls between individual Campbell's monkeys (Lemasson et al. 2011) and rhesus macaques (*Macaca mulatta*) (Pfefferle et al. 2016), for example, have been reported to be independent of genetic relatedness, but positively associated with social familiarity (measured by the duration of grooming each other and group-matrilineal membership, respectively). On the other hand, contact calls of mandrills (Levréro et al. 2015) and Tonkean macaques (*Macaca tonkeana*) (De Marco et al. 2019) have been found to be more similar acoustically between individuals with higher degrees of both genetic relatedness and social familiarity (measured by the length of time co-resident in the same group and group membership, respectively).

The golden snub-nosed monkey (*Rhinopithecus roxellana*), a species of colobine monkey endemic to China, inhabits temperate forests in mountainous areas at high altitudes of 1,000–4,100 m (Kirkpatrick and Grueter 2010). Breeding is strictly seasonal, with a mating peak occurring between September and November, and almost all birth events between March and May (Ren et al. 2003; Li and Zhao 2007). Golden snub-nosed monkeys are well-known for their large multilevel societies, rare among primates, composed of several one-male multi-female units (OMUs) (these OMUs collectively are also called a breeding band) and one (occasionally more than one) all-male unit (AMU) (Grueter et al. 2020; Qi et al. 2023). While the social units of a given group maintain a close association, each unit is distinct spatially and socially: the individuals of the same unit usually stay much closer to each other than to those of other units, and social interactions occur much more frequently among individuals within than across units (about 75% vs. 25%) (Zhang et al. 2008, 2012; Wang et al. 2013; Wada et al. 2015). The adult male resident in an OMU attempts to monopolize mating opportunities within the OMU, while about 30% of adult females copulate with and 57% of infants are sired by extra-unit adult males (i.e., adult males from other OMUs or the AMU) (Qi et al. 2020; Xiang et al. 2022). Infants and younger juveniles of similar age from different OMUs play frequently with each other (Li et al. 2011). Males can transfer between OMUs and the AMU within a group, and disperse between groups (Zhao and Li 2009; Yao et al. 2011; Xiang et al. 2014; Qi et al. 2017). Similarly, females can also transfer between OMUs within a group and disperse between groups (Qi et al. 2009; Zhang et al. 2012).

Kinship between adult females has been suggested to play a critically important role in the formation and stability of the multilevel social structure in golden snub-nosed monkeys (Guo et al. 2015; Ren et al. 2018; Fang et al. 2022). For instance, adult females within OMUs have been reported to be more genetically related than those across OMUs (Guo et al. 2015; Ren et al. 2018), and adult females have been observed to be more likely to disperse into OMUs containing their relatives (Guo et al. 2015). Furthermore, affiliative behaviors between adult females such as grooming each other have been reported to occur more frequently within than across OMUs (about 78% vs. 22%) (Zhang et al. 2008), and the frequency of occurrence of affiliative behaviors between adult females has been found to be positively associated with the degree of genetic relatedness (Ren et al. 2018).

Vocal communication is important for golden snub-nosed monkeys, particularly given that most group members are not visible to each other due to the large group size and closed forest habitat. However, systematic studies on the vocal communication of this primate have been lacking. A descriptive report on the vocal repertoire of adult golden snub-nosed monkeys has shown that *coo* calls (contact calls, hereafter), one call type most frequently produced, are the main vocalizations used to maintain contact and spacing at both levels within and across social units, and in various contexts including group movement, foraging, and resting (Fan et al. 2018). As in many other mammals (elephants: McComb et al. 2003; macaques: Sugiura 2007), contact calls of golden snub-nosed monkeys possess the properties of being tonal in structure and of relatively low frequency with few modulations (Fan et al. 2018, 2019). Taking advantage of the long-term demographic monitoring of a provisioned free-ranging group of golden snub-nosed monkeys in Shennongjia National Park, China, this study provided a systematic acoustic analysis for contact calls of adult females in relation to both kinship and social familiarity. It was predicted that structural similarities of contact calls between individuals increase with the degree of genetic relatedness (prediction 1) and/or the length of time co-resident in the same OMU (prediction 2), but decrease with age difference (prediction 3).

## Materials and methods

### Study site and subjects

This study was conducted in a provisioned free-ranging group of golden snub-nosed monkeys in the area around the Dalongtan Conservation Station (31°29′65″N, 110°17′93″E, 2,170 m) in Shennongjia National Park, Hubei Province, China (Yao et al. 2011). The topography within this area is extremely rugged with an elevational range of 2,000–2,700 m a.s.l. The climate is strongly seasonal. The mean monthly temperature is lowest in January (ca. −4 °C) and highest in July (ca. 17 °C). The annual amount of precipitation is ∼1,800 mm, with the rainy season from July to September. This area has a prolonged winter, and snowfalls usually last from November to March. The vegetation is characterized by deciduous broadleaf and evergreen conifer mixed forests.

The monkey group has been provisioned and completely habituated since January 2006 (Yao et al. 2011). The animals are provisioned 2 or 3 times per day with apples, carrots, oranges, peaches, and sweet potatoes, pine seeds, and lichens, among which the latter 2 are their most important natural foods (Liu et al. 2013). When not provisioned, the animals forage freely within an area of ∼9 km^2^ around the provisioning site. Close proximity (0.5–10 m) allows for to identify of all individuals, including their age and sex classes and unit memberships, based on their physical characteristics such as body size, hair coloration, face shape, canine size, scars, and genitalia (Yao et al. 2011). Group size and composition, including birth (e.g., mothers and the resident males), death, and dispersal events, have been counted continuously since January 2006 (Yao et al. 2011). During the periods of vocalization recording (January–February, July–December 2019; January, August–November 2020; January–February, October 2021; March–August 2022; March–September 2023; March–September 2024), a total of 152 different individuals had been present in the monkey group. At any time point, the group contained 69–90 individuals, including 20–31 adult females (≥5 years old), 6–23 adult males (≥7 years old), 32–48 juveniles (females: 1.5–5 years old; males: 1.5–7 years old) and infants (0–1.5 years old), forming 5–7 OMUs and 1 AMU. Across the whole period of vocalization recording, a total of 46 different OMUs (considered as a different OMU if the composition of adult individuals changed) were observed, and each OMU contained 1 adult male and 1–8 (mean: 4 ± SD 2) adult females. These adult females were selected as study subjects due to their importance in the maintenance of the multilevel social structure (Zhang et al. 2008, 2012; Wang et al. 2013).

### Vocalization recording and acoustic analysis

Vocalizations were recorded using a combination of focal and ad libitum sampling methods when the monkeys were under relaxed conditions and not disturbed by factors such as provisioning and ecotourism. Specifically, one adult female was selected as the focal animal on an observation day (08:00–18:00), and vocalizations including contact calls were recorded (in multiple 10-min sessions) using a Tascam DR44-WL digital recorder at a 44.1 kHz (16 bits) sampling rate, connected to a Sennheiser ME66 directional microphone with a deadcat furry windshield. Vocalization recording was then rotated to another adult female the next day, and attempts were made to rotate evenly among all adult females present. Calls of non-focal adult females were also recorded opportunistically to increase the total number of vocalization samples. The distance between the microphone and callers was kept within 1–5 m. For each recorded vocalization, the concurrent contextual information was noted by speaking into a lapel microphone using the second audio channel of the recording equipment, complementarily by videotaping with a digital camera by a field assistant (HDR-XR 260, Sony, Japan).

For each 10-min vocalization file, narrow-band spectrograms were generated using the Praat 6.1.56 package (Gaussian window shape, view range = 0–20 kHz, window length = 0.03 s, dynamic range = 50 dB) (Boersma P and Weenink D, University of Amsterdam, the Netherlands; Fan et al. 2018). Based on auditory sense and visual inspection of spectrograms, the vocalizations with excessive background noise or overlapped by other calls were excluded, and only the contact call recordings of high quality from known adult female callers were selected for further acoustic analysis. Ultimately, a total of 1,071 contact calls from 31 individuals were selected, with 25–82 (mean: 35 ± SD 12) calls from each individual. For each selected recording, a total of 35 temporal, spectral, and amplitude parameters were extracted to describe the acoustic structure via Praat 6.1.56. The points on the fundamental frequency contour that jumped abnormally were adjusted manually. These parameters and their definitions, and the extraction methods are listed in Table 1.

**Table 1:** Acoustic parameters and their definitions of contact calls from adult female golden snub-nosed monkeys.

Parameter	Definition (unit)
Duration	Duration of the entire call (s)
%T_min*f*_0_	Percentage of the duration from the start to minimum fundamental frequency out of entire call duration (%)
%T_max*f*_0_	Percentage of the duration from the start to maximum fundamental frequency out of entire call duration (%)
%T_minA	Percentage of the duration from the start to minimum amplitude out of entire call duration (%)
%T_maxA	Percentage of the duration from the start to maximum amplitude out of entire call duration (%)
*f*_0__start	Fundamental frequency at the start of the call (Hz)
*f*_0__end	Fundamental frequency at the end of the call (Hz)
*f*_0__min	Minimum fundamental frequency of the entire call (Hz)
*f*_0__max	Maximum fundamental frequency of the entire call (Hz)
*f*_0__range	Difference between minimum and maximum fundamental frequencies of the entire call (Hz)
*f*_0__Q_1_	First quartile of fundamental frequency of the entire call (Hz)
*f*_0__Q_2_	Second quartile or median of fundamental frequency of the entire call (Hz)
*f*_0__Q_3_	Third quartile of fundamental frequency of the entire call (Hz)
*f*_0__IQR	Interquartile range of fundamental frequency of the entire call (Hz)
*f*_0__SD	Standard deviation of fundamental frequency of the entire call (Hz)
*f*_0__mean	Mean of fundamental frequency of the entire call (Hz)
*F*_1_	First formant frequency of the entire call (Hz)
*F*_2_	Second formant frequency of the entire call (Hz)
*F*_3_	Third formant frequency of the entire call (Hz)
*F*_4_	Fourth formant frequency of the entire call (Hz)
Δ*F*	Average difference between neighboring formant frequencies (i.e., formant dispersion) of the entire call (Hz)
A_start	Amplitude at the start of the call (dB)
A_end	Amplitude at the end of the call (dB)
A_min	Minimum amplitude of the entire call (dB)
A_max	Maximum amplitude of the entire call (dB)
A_range	Difference between minimum and maximum amplitudes of the entire call (dB)
A_Q_1_	First quartile of amplitude of the entire call (dB)
A_Q_2_	Second quartile or median of amplitude of the entire call (dB)
A_Q_3_	Third quartile of amplitude of the entire call (dB)
A_IQR	Interquartile range of amplitude of the entire call (dB)
A_SD	Standard deviation of amplitude of the entire call (dB)
A_mean	Mean amplitude of the entire call (dB)
HNR	Harmonics-to-noise ratio: periodic distribution of energy across the entire call (dB)
Jitter	Cycle-to-cycle variability of fundamental frequency across the entire call (%)
Shimmer	Cycle-to-cycle variability of amplitude across the entire call (%)

Using the command (sound: to pitch [cc]; time step = 0.01 s, pitch floor = 75 Hz, pitch ceiling = 1,200 Hz) to determine the time points for the start and end of the call, and the time points for the minimum and maximum fundamental frequencies, to calculate Duration, %T_min*f*_0_, and %T_max*f*_0_.

Using the command (sound: to pitch [cc]; time step = 0.01 s, pitch floor = 75 Hz, pitch ceiling = 1,200 Hz) to extract *f*_0__start, *f*_0__end, *f*_0__min, *f*_0__max, *f*_0__mean, *f*_0__SD, *f*_0__Q_1_, *f*_0__Q_2_, *f*_0__Q_3_ and *f*_0__IQR; *f*_0__range was calculated as *f*_0__max – *f*_0__min.

Using the command (sound: to intensity; time step = 0.008 s, minimum pitch = 100 Hz) to determine the time points for the minimum and maximum amplitudes to calculate %T_minA, and %T_maxA.

Using the command (sound: to intensity; time step = 0.008 s, minimum pitch = 100 Hz) *t*0 extract A_start, A_end, A_min, A_max, A_mean, A_SD, A_Q_1_, A_Q_2_, A_Q_3_ and A_IQR; A_range was calculated as A_max – A_min.

Using the command (analyze: to formant(burg); time step = 0.00625 s, max number of formants = 5, maximum formant = 8,000 Hz, window length = 0.025) *t*0 extract *F*_1_, *F*_2_, *F*_3_, *F*_4_, and Δ*F*.

Using the command (pulse: voice report) to extract HNR, Jitter, and Shimmer.

To estimate structural similarities of calls between individuals, a stepwise discriminant analysis was employed to generate the pairwise *F* values. The smaller the *F* value was, the more similar the calls of 2 individuals were, and vice versa. This approach has been applied in some other studies on animal vocal communication (Thinh et al. 2011; Meyer et al. 2012; Pfefferle et al. 2016). In the discriminant analysis, some parameters were arcsine (%T_min*f*_0_, %T_max*f*_0_, %T_minA, %T_maxA), square root (*f*_0__min and A_start), or logarithmic (*f*_0__start, *f*_0__end, *f*_0__max, *f*_0__range, *f*_0__IQR, *f*_0__SD, *f*_0__mean, and F_1_) transformed to approximate normality, verified by the normal Q–Q plot. The method of Wilk's λ and the selection criteria of *P* values (entry: *P* ≤ 0.05; removal: *P* ≤ 0.10) were chosen. Since data sets were unbalanced, prior probabilities and classification coefficients were adjusted according to the observed group sizes (i.e., the call number from each individual). The *leave-one-out* classification method was employed for the cross-validation. A binomial test (2-tailed and with a significance level of 0.05) was used to examine whether the classification accuracy was better than expected by chance. All analyses were done using SPSS 21.0 (Armonk, NY, USA: IBM Corp.).

### Determination of kinship

Hair samples were collected for the analysis of kinship between individual callers. When collecting hair samples from a target individual, 1 field assistant used the preferred foods (e.g., peanuts) to attract its attention, while another assistant wore sterile rubber gloves, approached the animal from behind, waited for appropriate opportunities to pluck hairs, and immediately put them in a 50-mL centrifuge tube. At least 100 hairs with follicles were collected from each individual, stored at −20 °C in the field, and sent to the laboratory within 1 week for subsequent genetic analysis. DNA was extracted from the hair follicles using the TIANGEN magnetic bead method (universal genomic DNA extraction kit), and then DNA libraries were constructed using the Rapid Plus DNA Lib Prep Kit for Illumina, followed by whole genome sequencing using DNBSEQ-T. Germline short variants were analyzed and filtered using Genome Analysis Toolkit (GATK) Haplotype Caller and GATK Variant Filter, respectively, to identify single-nucleotide polymorphisms and small insertion-deletions (Indels). Finally, the coefficients of relatedness (*r*) were determined using PLINK v2.00a5.10LM and King 2.3.2 and confirmed complementarily by the previous pedigree record. With reference to other studies on the relationship between genetic and acoustic similarities (Levréro et al. 2015; Pfefferle et al. 2016), the kin relationships between 2 individuals in this study were classified as nonkin (*r* < 0.125), distant kin (0.125 ≤ *r* < 0.25), and close kin (*r* ≥ 0.25). The numbers of individual pairs were 114, 188, and 163 for nonkin, distant kin, and close kin, respectively (a total of 465 individual pairs could be constituted by 31 individuals). For each individual, nonkin, distant kin, and close kin accounted for 26.0 ± SD 25.0%, 38.1 ± 17.7%, and 35.9 ± 16.9% of other individuals, respectively. In terms of the distribution across OMUs during the period of vocalization recording, for each individual, nonkin, distant kin, and close kin accounted for 23.9 ± 30.3% vs. 27.5 ± 26.3%, 33.6 ± 27.3% vs. 38.3 ± 20.0%, and 42.4 ± 29.7% vs. 34.1 ± 17.8% of other individuals within its own OMU vs. from other OMUs, respectively. These proportions of individual numbers belonging to different kinship categories did not differ between within and across OMUs (Pearson *χ*^2^ test: *χ*^2^ = 1.372, *df* = 2, *P* = 0.504).

### Assessment of familiarity

As mentioned above, social (especially affiliative) interactions among adult female golden snub-nosed monkeys occur much more frequently within than across OMUs (Zhang et al. 2008, 2012; Wang et al. 2013). Thus, 2 individuals were assumed to be more familiar if they co-resided in the same OMU for a longer time. The cumulative length of time, from the date when they began to co-reside in the same OMU (after birth or immigrating into the group) to the date when the recording of their vocalizations was ended, was used as an index of social familiarity between 2 individuals. In this study, the length of time co-resident in the same OMU (converted into the number of months) were classified into 4 categorical levels: never (= 0 month), short (>0, ≤18 months), medium (>18, ≤72 months), and long (>72 months). The longest time co-resident in the same OMU of 2 individuals was 192.4 months. The primary consideration of this categorization method was to obtain comparable numbers of individual pairs across different levels for a high level of effectiveness in statistical analysis. Thus, despite the fact that 289 out of 465 individual pairs never co-resided in the same OMU, the numbers of individual pairs were balanced relatively well across the other 3 levels: 55 for short, 66 for medium, and 55 for long, respectively.

In golden snub-nosed monkeys, infants and younger juveniles of similar age frequently play and preferentially interact with each other (Li et al. 2011), as mentioned above. Two individuals were assumed to be more familiar if their ages were more proximate, and thus, the age difference between individuals was used as another indicator of familiarity, as suggested in other studies (Widdig et al. 2001; Pfefferle et al. 2013; Levréro et al. 2019). The calculation of the age difference between 2 individuals was straightforward, just converting the number of days between their birth dates to the number of years.

### Statistical analysis

A linear mixed model was fitted to analyze structural similarities of contact calls (i.e., the *F* values generated by the stepwise discriminant analysis) in relation to kinship and social familiarity (i.e., the length of time co-resident in the same OMU and age difference) between individuals. The model was fitted in R 4.4.1 (R Core Team 2024) with the function “lmer” provided in the package “lme4” (Bates et al. 2015). Kinship and the length of time co-resident in the same OMU were considered as 2 categorical predictors and dummy coded, while age difference was regarded as a continuous predictor and was square root transformed to approximate normality, verified by a Q–Q plot generated by the function “qqnorm.” The *F* values, the continuous dependent variable, were logarithmically transformed to approximate the normal distribution, verified by a Q–Q plot (the normality of residuals was verified as well). To examine the potential effects of interaction terms among the main predictors, the 3-way and all 2-way interaction terms were included in the full model. As random factors, the identities of 2 individual callers were always included in the model. Collinearities among the main predictors were checked using the function “vif” in the package “car” (Fox and Weisberg 2019) for the model excluding interaction terms. Results indicated that a collinearity issue did not exist (generalized variance inflation factors: 1.01–1.08). Heavily influential cases did not exist either, confirmed by comparing the model estimates based on the data excluding one case at a time with the estimates based on all data points.

The overall effects of all main predictors and their interaction terms were examined by comparing the full model and the null model (including the intercept and random factors only), using a log likelihood ratio test by the function “anova” with the argument of test = “Chisq.” It turned out that the full model was significant (Supplementary Table S1). The significance of the 3-way interaction term and each of 2-way interaction terms were then examined, respectively, using the same approach. All interaction terms were successively removed from the full model due to insignificance (Supplementary Table S1). Finally, the effect of each main predictor was also analyzed using the same approach. The mean (and 95% confidence interval [CI]) of logarithmic transformed *F* values for each level of each categorical predictor was calculated using the function “effect” in the package “effects” (Fox and Weisberg 2019). If a categorical predictor had a significant effect, post hoc pairwise comparisons were conducted using the Tukey test by the function “glht” in the package “multcomp” (Hothorn et al. 2008). All tests were 2-tailed with a significance level of 0.05.

## Results

### Acoustic similarity

The stepwise discriminant analysis correctly assigned 64.1% (cross-validation: 50.9%) of contact calls to their original callers, much better than 3.2% (i.e., 1/31) expected by chance (*P* < 0.001), indicating clear individuality in the acoustic structure. The first 6 canonical discriminant functions could explain 80% of the variance, among which the first one could explain 54% of the variance (Supplementary Table S2). The most important acoustic parameter to discriminate among individual callers was the mean fundamental frequency, followed by the range, maximum, and the third quartile values of the fundamental frequency (Supplementary Table S2).

**Figure 1:**
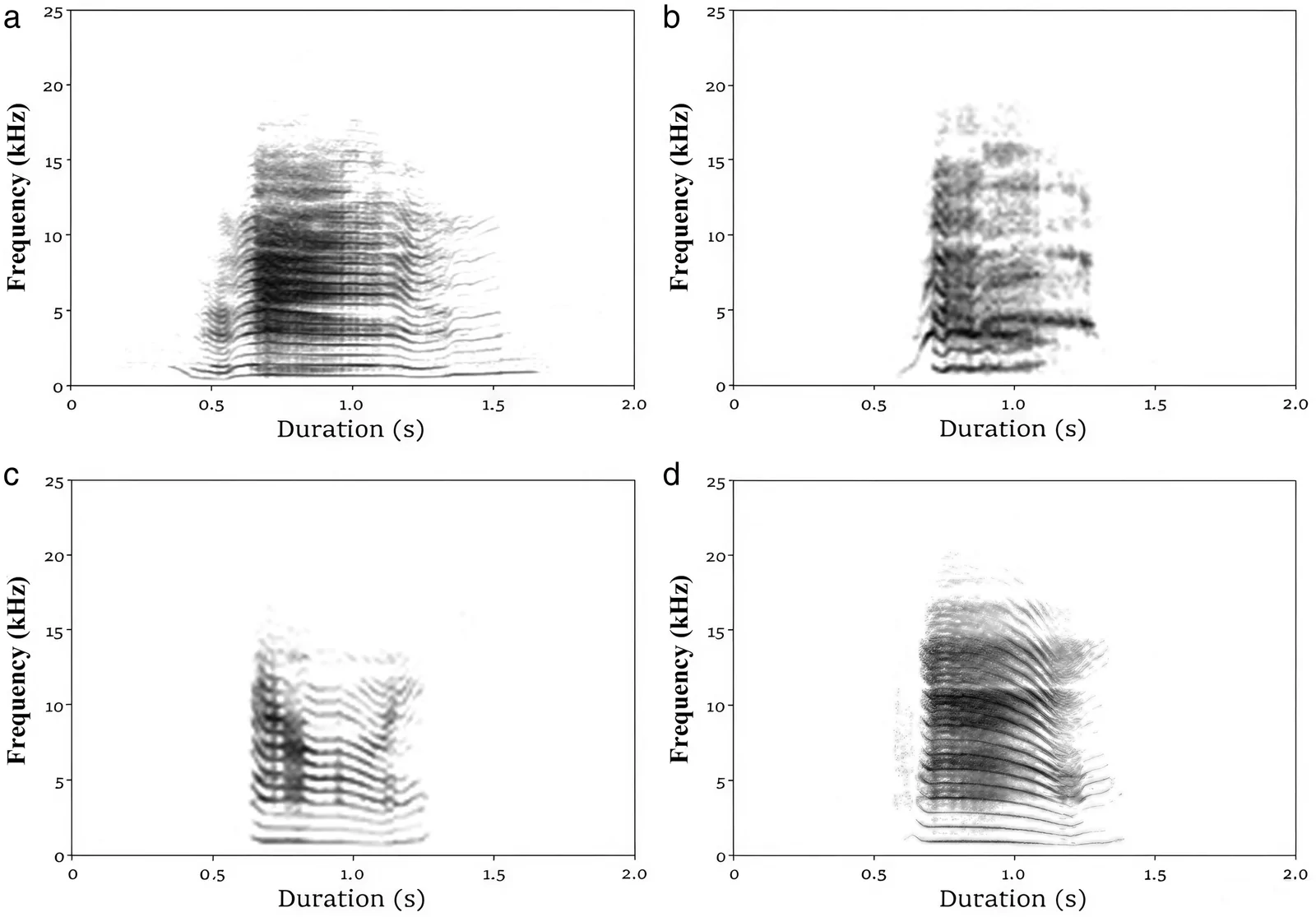
Spectrographic examples of contact calls with most dissimilar (a vs. b) and most similar (c vs. d) acoustic structures between individuals of adult female golden snub-nosed monkeys.

The *F* values (and logarithmic transformed values) generated by the stepwise discriminant analysis had a mean of 11.470 ± SD 7.893 (2.230 ± 0.649) for the 465 pairs of individual callers (Supplementary Figure S1). The maximum and minimum *F* values were 48.741 (3.887) and 1.722 (0.543), indicating that the structure of contact calls was most dissimilar (Fig. 1a,b) and most similar (Fig. 1c,d) between 2 individual callers, respectively, in terms of the acoustic parameters identified by the stepwise discriminant analysis (Supplementary Table S2).

### Effects of kinship and familiarity

**Figure 2:**
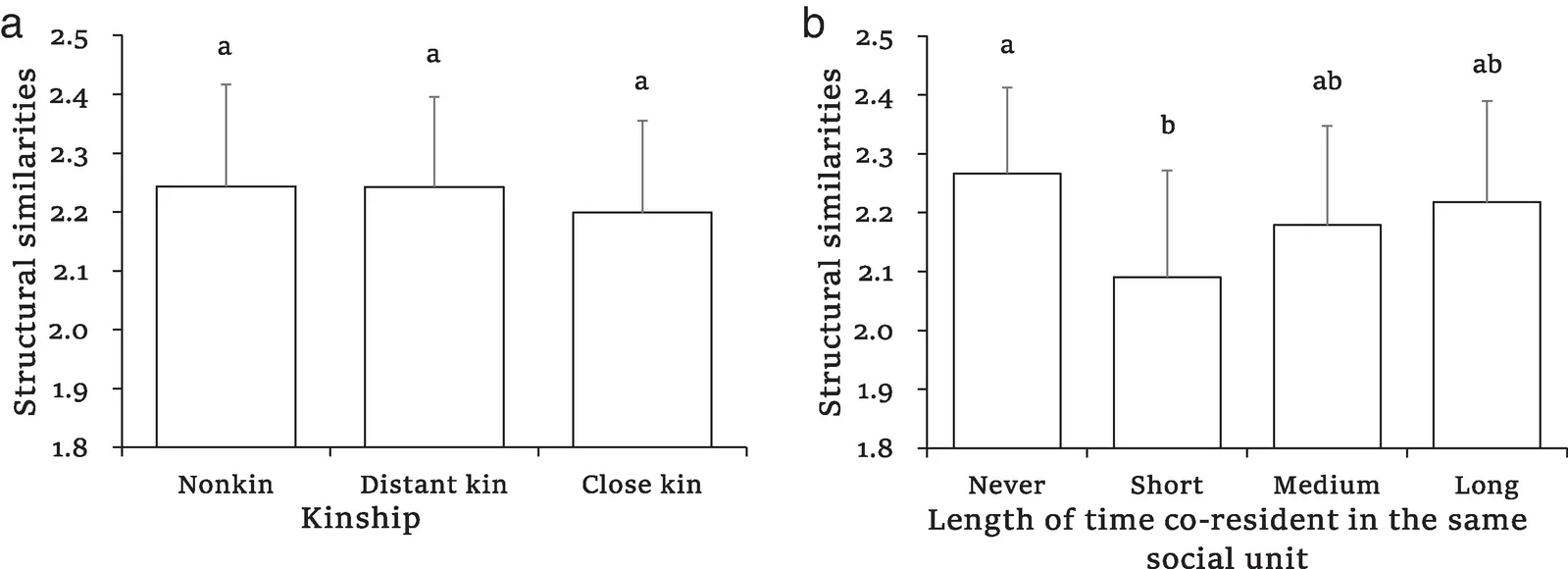
Structural similarities of contact calls between individuals of adult female golden snub-nosed monkeys (a) with different kin relationships and (b) co-residing in the same social unit for different lengths of time. Structural similarities were expressed as the mean and 95% CI of logarithmic transformed *F* values generated by a stepwise discriminant analysis. No difference was found among nonkin (*N* = 114 individual pairs with 31 individuals involved), distant kin (*N* = 188 individual pairs with 30 individuals involved), or close kin (*N* = 163 individual pairs with 30 individuals involved) based on a log likelihood ratio test with a significance level of 0.05. The calls were more similar if 2 individuals co-resided in the same unit for a short time (>0, ≤18 months) (*N* = 289 individual pairs with 31 individuals involved) than being never co-resident (*N* = 55 individual pairs with 31 individuals involved) based on the Tukey test for post hoc pairwise comparisons (*P* < 0.05). No other pairwise differences were detected between different lengths of time of co-residence (medium: >18, ≤72 months; *N* = 66 individual pairs with 30 individuals involved) (long: >72 months; *N* = 55 individual pairs with 31 individuals involved). Same letters indicate no significant differences.

No significant effect was detected for kinship on structural similarities of contact calls between individuals (*χ*^2^ = 1.002, *df* = 2, *P* = 0.606) (Table 2). The means (95% CI) of logarithmic transformed *F* values were 2.243 (2.070–2.416) for nonkin, 2.243 (2.090–2.396) for distant kin, and 2.199 (2.043–2.355) for close kin, after controlling for the length of time co-resident in the same OMU and age difference (Fig. 2a).

**Table 2:** Effects of kinship, length of time co-resident in the same social unit, and age difference on structural similarities of contact calls between individuals of female golden snub-nosed monkeys.

	Estimate	SE	*t*-value
(Intercept)	1.477	0.098	15.063
Distant kin	−0.001	0.075	−0.008
Close kin	−0.044	0.079	−0.561
Short time	−0.176	0.067	−2.610
Medium time	−0.088	0.058	−1.512
Long time	−0.049	0.059	−0.833
Age difference	0.402	0.022	18.582

Log likelihood ratio test: kinship (*χ*^2^ = 1.002, *df* = 2, *P* = 0.606), length of time co-resident in the same unit (*χ*^2^ = 8.055, *df* = 3, *P* = 0.045), age difference (*χ*^2^ = 260.090, *df* = 1, *P* < 0.001).

Tukey test for pairwise comparisons among different lengths of time co-resident in the same unit: never vs. short (*z* = 2.610, *P* = 0.043), never vs. medium (*z* = 1.512, *P* = 0.420), never vs. long (*z* = 0.833, *P* = 0.833), short vs. medium (*z* = 1.092, *P* = 0.686), short vs. long (*z* = 1.532, *P* = 0.408), medium vs. long (*z* = 0.528, *P* = 0.950).

However, structural similarities of the calls between individuals varied significantly among different lengths of time co-resident in the same OMU (*χ*^2^ = 8.055, *df* = 3, *P* = 0.045) (Table 2). Specifically, the calls were more similar between individuals co-resident in the same OMU for a short time (i.e., up to 18 months) than those between individuals never co-resident in the same OMU (*z* = 2.610, *P* = 0.043). No other pairwise differences were found between different lengths of time co-resident in the same OMU (never vs. medium: *z* = 1.512, *P* = 0.420; never vs. long: *z* = 0.833, *P* = 0.833; short vs. medium: *z* = 1.092, *P* = 0.686; short vs. long: *z* = 1.532, *P* = 0.408; medium vs. long: *z* = 0.528, *P* = 0.950). The mean (95% CI) of logarithmic transformed *F* values was 2.267 (2.121–2.412) for 2 individuals never co-resident in the same OMU, while the values were 2.091 (1.910–2.272), 2.179 (2.010–2.347), and 2.218 (2.046–2.390) for individuals co-resident for a short, medium, and long time, respectively, after controlling for kinship and age difference (Fig. 2b).

**Figure 3:**
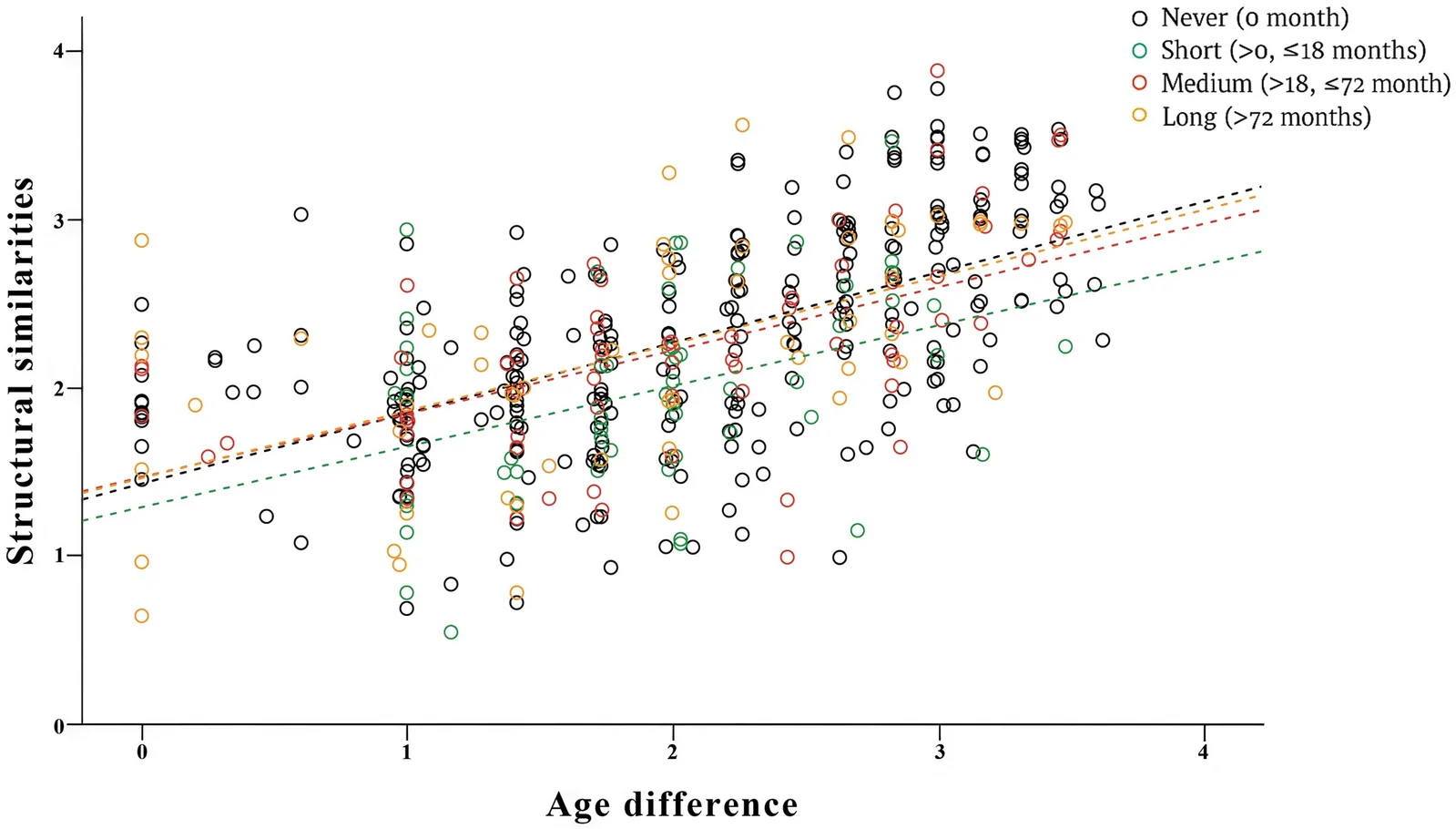
Scatterplot of structural similarities of contact calls and age differences between individuals of adult female golden snub-nosed monkeys. Structural similarities were expressed as logarithmic transformed *F* values generated by a stepwise discriminant analysis, and shown separately by different colors for different lengths of time co-resident in the same social unit (*N* = 289, 55, 66, and 55 individual pairs with 31, 31, 30, and 31 individuals involved for never, short, medium, and long, respectively). Age differences were square root transformed.

In addition, age difference clearly affected structural similarities of the calls between individuals (*χ*^2^ = 260.090, *df* = 1, *P* < 0.001) (Table 2). The more proximate the ages of 2 individuals were, the more similar in the structure of their calls were (Fig. 3). After the influence of kinship was ignored due to insignificance, the linear trend that structural similarities of contact calls varied with age differences between individuals appeared to be very similar among different lengths of time co-resident in the same OMU. The linear regression coefficients were 0.419, 0.360, 0.377, and 0.399, respectively, which were not different statistically (analysis of covariance: *F* = 0.162, *df*_1_ = 3, *df*_2_ = 457, *P* = 0.922) (Zar 1999).

## Discussion

This study presented the first acoustic analysis for contact calls of adult female golden snub-nosed monkeys, with the consideration of both kinship and social familiarity. Contrary to prediction 1, no significant difference was detected in structural similarities of the calls between individuals among nonkin, distant kin, or close kin. The mechanism of kin discrimination by acoustic phenotype matching was not supported. Similar findings have also been reported in studies of some primates such as Japanese macaques (*Macaca fuscata*: Tanaka et al. 2006), rhesus macaques (Pfefferle et al. 2016), Campbell's monkeys (Lemasson et al. 2011), chimpanzees (*Pan troglodytes*: Marshall et al. 1999; Crockford et al. 2004), and bonobos (*Pan paniscus*: Levréro et al. 2019). On the other hand, this result was not consistent with the findings of some other studies (e.g., mandrills: Levréro et al. 2015; Tonkean macaques: De Marco et al. 2019), in which structural similarities of contact calls between individuals have been observed to increase with the degree of genetic relatedness after controlling for social familiarity.

This observation that structural similarities of contact calls between individuals of adult female golden snub-nosed monkeys were independent of the degree of genetic relatedness could be attributed to the following 3 factors. First, acoustic features encoding kinship information may have been overlooked. However, this possibility is likely minimal, given that a large number (i.e., 35) of parameters related to temporal, spectral, and amplitude features were used to describe the global structure of vocalizations in this study. Different types of information have been suggested to be reflected by different acoustic parameters of vocalizations (Snowdon and Cleveland 1980; Elowson and Snowdon 1994; Levréro et al. 2015). A study on the effects of kinship and familiarity on structural similarities of contact calls between individual mandrills has reported, for instance, that the mean fundamental frequency is more tightly linked to genetic relatedness, whereas the standard deviation of the fundamental frequency is more plastic and more likely to be influenced by social familiarity (Levréro et al. 2015). In fact, previous studies on the relationship between acoustic and genetic similarities have used different approaches to describe the acoustic features (Levréro et al. 2015; Pfefferle et al. 2016; De Marco et al. 2019), and this issue even exists in different studies of the same species (Pfefferle et al. 2016; De Marco et al. 2019). For example, a total of 112 acoustic parameters were used to describe the global structure of contact calls in a study of rhesus macaques (Pfefferle et al. 2016), whereas only 7 parameters were used as the representative of the acoustic structure in a study of mandrills (Levréro et al. 2015). However, the potential impacts of different approaches to describe the acoustic structure of vocalizations on research results have seldom been evaluated. Thus, whether adult female golden snub-nosed monkeys could use contact calls to assess kin relationships to each other still needs to be elucidated by subsequent playback experiments.

Second, adult female golden snub-nosed monkeys emit contact calls in different behavioral contexts, including moving, resting, and foraging (Fan et al. 2018, 2019). The acoustic structure of vocalizations from the same individual may vary among different contexts in which vocalizations are produced, as suggested in other studies (Boinski and Mitchell 1997; Slocombe and Zuberbühler 2006; Sugiura 2007). However, the effects of possible within-individual variation in the acoustic structure could not be excluded in this study, which would require recording many more vocalizations from each individual.

Third, it is likely that acoustic features encoding kinship information were masked in this study by vocal accommodation (convergence and/or divergence). This point is supported by the nonlinear relationship between acoustic similarities and social familiarity between individuals, detected in this study (discussed below). As mentioned above, like other social animals, a group of golden snub-nosed monkeys contains related individuals, as well as unrelated ones with which an individual interacts regularly and repeatedly (Cheney and Seyfarth 1982; Rebout et al. 2021). In particular, group members are distributed in several distinct social units (Grueter et al. 2020; Qi et al. 2023), and an adult female needs to interact with other females from not only their own but also other OMUs (Zhang et al. 2008). Since most group members are not visible to each other, acoustic signals are particularly critical for social communication among individuals, and indeed contact calls have recently been reported to play important roles in mediating the complex interactive network within multilevel societies of this primate species (Yang et al. 2024). What's more, it has been suggested that a higher level of flexibility (as such less genetically determined) should occur in calls of higher social values (e.g., contact calls of golden snub-nosed monkeys) that require more adaptation to the calls of specific interactive partners than in calls of lower social values targeting the whole group (e.g., alarm calls) (Snowdon and Hausberger 1997; Riede 2010; Lemasson and Hausberger 2011).

Only partly consistent with prediction 2, contact calls of 2 individuals of adult female golden snub-nosed monkeys converged first and then exhibited a tendency to diverge as the length of time co-resident in the same OMU increased, while the acoustic structure exhibited clear individuality. Specifically, as predicted, if 2 individuals co-resided in the same OMU for a period of up to 18 months, structural similarities of their contact calls increased significantly, compared with those never co-residing in the same OMU. However, if 2 individuals co-resided in the same OMU for a period of longer than 18 months, structural similarities of their contact calls subsequently tended to decrease. Of course, the specific length of time co-resident in the same OMU associated with temporary vocal convergence needs to be elucidated in future studies.

On the one hand, golden snub-nosed monkeys live in large social groups with multiple levels (Grueter et al. 2020; Qi et al. 2023). Compared with animals living in small and single-layered groups, there should be more diverse social interactions among more individuals with differing roles and intentions, leading to increased uncertainty about callers' identities, and as such increased selection on callers to produce individualized calls (Tibbetts and Dale 2007; Pollard and Blumstein 2011; Carlson et al. 2020; Smith-Vidaurre et al. 2021). Indeed, the results of both this study and previous ones (Fan et al. 2019; Yang et al. 2024) showed that contact calls of golden snub-nosed monkeys exhibited clear individuality. Some other species of mammals living in groups with social structures similar to that of golden snub-nosed monkeys have also been observed to produce contact calls with high levels of individuality to maintain affiliative contacts and interactions with conspecifics, including African elephants (*Loxodonta africana*: McComb et al. 2000, 2003; Soltis et al. 2005), common bottlenose dolphins (*Tursiops truncatus*: Janik and Slater 1998; Tyack 2000), and Indo-Pacific bottlenose dolphins (King et al. 2018).

The capability of individual discrimination based on contact calls in golden snub-nosed monkeys awaits to be confirmed by further studies. Provided that individuals could be accurately discriminated, the receivers might assess kinship to the callers by associating the callers' identities with the knowledge of the callers' kin relationships based on other call types or even another domain (e.g., visual), as suggested in several studies (Tang-Martinez 2001; Kondo and Watanabe 2009; Pfefferle et al. 2016; Keenan et al. 2020; Mine et al. 2024).

On the other hand, while vocal convergence to signal social affiliation and closeness has been observed repeatedly (“social badge” signals) (greater spear-nosed bats, *Phyllostomus hastatus*: Boughman 1997; pygmy marmosets, *Cebuella pygmaea*: Snowdon and Elowson 1999; chimpanzees: Crockford et al. 2004; humans, *Homo sapiens*: Pardo 2006; Campbell's monkeys: Lemasson et al. 2011; mandrills: Levréro et al. 2015; rhesus macaques: Pfefferle et al. 2016; Tonkean macaques: De Marco et al. 2019; reviewed in Ruch et al. 2018), temporary vocal convergence during the period of instability of social bonds or group cohesion have been reported in only a few species among mammals, including pygmy marmosets (Elowson and Snowdon 1994), Wied's marmosets (*Callithrix kuhlii*: Rukstalis et al. 2003), Campbells' monkeys (Lemasson and Hausberger 2004), and African elephants (Poole et al. 2005). It has been suggested that this temporary pattern of vocal accommodation is likely to reflect the increased need to enhance social bonding in a pair and the cohesiveness of groups (Lemasson and Hausberger 2004; Poole et al. 2005; Tyack 2008). This could explain the result of this study well, regardless of whether the 2 periods of same-OMU co-residence and vocalization recording overlapped completely or not. Perhaps, once the degree of social bonding and familiarity between 2 individuals reached a threshold, the corresponding pressure relaxed and then the calls diverged individually again. Thus, the acoustic structure of contact calls of adult female golden snub-nosed monkeys was likely to reflect a dynamic tradeoff between maintaining social bonds and advertising individual identities (Zürcher et al. 2021). The dependence of structural modifications in acoustic signals on social dynamics suggests that vocal accommodation should not be simply considered as a result of relative auditory exposure (Ruch et al. 2018; Langehennig-Peristenidou and Scheumann 2024; Painter et al. 2024).

Consistent with prediction 3, structural similarities of contact calls between individuals decreased with age differences in adult female golden snub-nosed monkeys. The negative relationship between acoustic similarities and age differences has also been observed in contact calls of rhesus macaques (Pfefferle et al. 2016) and bonobos (*Pan paniscus*: Levréro et al. 2019). Familiarity and social bonding could be a possible explanation (Smith et al. 2003; Widdig 2007; Levréro et al. 2019). The high level of individuality in the calls, however, made another explanation more plausible, that is, age-related morphological changes in the vocal apparatus (Ey et al. 2007; da Silva et al. 2011; Kreiman and Lee 2025).

In summary, this study did not find evidence that kinship information was encoded in the acoustic structure of contact calls of adult female golden snub-nosed monkeys. However, the calls between individuals became more similar first and then dissimilar again as the length of time co-resident in the same OMU increased, reflecting the importance of temporary vocal convergence in enhancing social bonding and familiarity. The results of this study improved our understanding of the functions of these vocalizations more specifically and provided a basis for investigating the mechanisms of kin discrimination via acoustic signals and, in turn, ultimately improved the understanding of the maintenance mechanism of the multilevel social structure in this primate. In addition, the potential of kin discrimination based on contact calls still needs to be elucidated in future studies, such as playback experiments with the effects of familiarity being better controlled for, especially given that all subjects were from the same group in this study.

## Supplementary Material

zoaf060_Supplementary_Data

## References

[zoaf060-B1] Bates D, Mächler M, Bolker B, Walker S, 2015. Fitting linear mixed-effects models using lme4. J Stat Softw 67(1):1–48.

[zoaf060-B2] Bohn KM, Wilkinson GS, Moss CF, 2007. Discrimination of infant isolation calls by female greater spear-nosed bats, *Phyllostomus hastatus*. Anim Behav 73(3):423–432. 18311319 10.1016/j.anbehav.2006.09.003 PMC2000849

[zoaf060-B3] Boinski S, Mitchell CL, 1997. Chuck vocalizations of wild female squirrel monkeys (*Saimiri sciureus*) contain information on caller identity and foraging activity. Int J Primatol 18(6):975–993.

[zoaf060-B4] Boughman JW , 1997. Greater spear-nosed bats give group-distinctive calls. Behav Ecol Sociobiol 40(1):61–70.

[zoaf060-B5] Carlson NV, Kelly EM, Couzin L, 2020. Individual vocal recognition across taxa: a review of the literature and a look into the future. Philos Trans R Soc Lond B Biol Sci 375(1802):20190479. 32420840 10.1098/rstb.2019.0479 PMC7331019

[zoaf060-B6] Charrier I, Mathevon N, Jouventin P, 2003. Vocal signature recognition of mothers by fur seal pups. Anim Behav 65(3):543–550.

[zoaf060-B7] Chaverri G, Araya-Ajoy YG, Sagot M, 2020. Contact calling in context: intra- and intergroup variation in vocalization rates depend on a call's function. Behav Ecol Sociobiol 74(5):57.

[zoaf060-B8] Cheney DL, Seyfarth RW, 1982. Recognition of individuals within and between groups of free-ranging vervet monkeys. Am Zool 22(3):519–529.

[zoaf060-B9] Crockford C, Herbinger I, Vigilant L, Boesch C, 2004. Wild chimpanzees produce group-specific calls: a case for vocal learning. Ethology 110(3):221–243.

[zoaf060-B10] da Silva PT, Master S, Andreoni S, Pontes P, Ramos LR, 2011. Acoustic and long-term average spectrum measures to detect vocal aging in women. J Voice 25(4):411–419. 20817474 10.1016/j.jvoice.2010.04.002

[zoaf060-B11] De Marco A, Rebout N, Massiot E, Sanna A, Sterck EHM et al, 2019. Differential patterns of vocal similarity in tolerant and intolerant macaques. Behaviour 156(12):1209–1233.

[zoaf060-B12] Elowson AM, Snowdon CT, 1994. Pygmy marmosets, *Cebuella pygmaea*, modify vocal structure in response to changed social environment. Anim Behav 47(6):1267–1277.

[zoaf060-B13] Ey E, Pfefferle D, Fischer J, 2007. Do age- and sex-related variations reliably reflect body size in non-human primate vocalizations? A review. Primates 48(4):253–267. 17226064 10.1007/s10329-006-0033-y

[zoaf060-B14] Fan P, Liu R, Grueter CC, Li F, Wu F et al, 2019. Individuality in coo calls of adult male golden snub-nosed monkeys (*Rhinopithecus roxellana*) living in a multilevel society. Anim Cogn 22(1):71–79. 30460512 10.1007/s10071-018-1222-y PMC6326966

[zoaf060-B15] Fan P, Liu X, Liu R, Li F, Huang T et al, 2018. Vocal repertoire of free-ranging adult golden snub-nosed monkeys (*Rhinopithecus roxellana*). Am J Primatol 80(6):e22869. 29767431 10.1002/ajp.22869 PMC6032912

[zoaf060-B16] Fang G, Jiao H, Wang M, Huang P, Liu X et al, 2022. Female demographic changes contribute to the maintenance of social stability within a primate multilevel society. Anim Behav 192:101–108.

[zoaf060-B17] Fischer J, Farnworth MS, Sennhenn-Reulen H, Hammerschmidt K, 2017. Quantifying social complexity. Anim Behav 130:57–66.

[zoaf060-B18] Fitch WT, Hauser MD, 1995. Vocal production in nonhuman primates: acoustics, physiology, and functional constraints on “honest” advertisement. Am J Primatol 37(3):191–219. 31936952 10.1002/ajp.1350370303

[zoaf060-B19] Fox J, Weisberg S, 2019. An R Companion to Applied Regression. 3^rd^ edn. Thousand Oaks (CA): Sage.

[zoaf060-B20] Greenwood PJ , 1980. Mating systems, philopatry and dispersal in birds and mammals. Anim Behav 28(4):1140–1162.

[zoaf060-B21] Grueter CC, Qi X, Zinner D, Bergman T, Li M et al, 2020. Multilevel organisation of animal sociality. Trends Ecol Evol 35(9):834–847. 32473744 10.1016/j.tree.2020.05.003

[zoaf060-B22] Guo S, Huang K, Ji W, Garber PA, Li B. 2015. The role of kinship in the formation of a primate multilevel society. Am J Phys Anthropol 156(4):606–613. 25448828 10.1002/ajpa.22677

[zoaf060-B23] Halpin ZT , 1991. Kin recognition cues of vertebrates. In: Hepper PG, editor. Kin Recognition. Cambridge: Cambridge University Press, 220–258.

[zoaf060-B24] Hamilton WD , 1964a. The genetical evolution of social behaviour. I. J Theor Biol 7(1):1–16. 5875341 10.1016/0022-5193(64)90038-4

[zoaf060-B25] Hamilton WD , 1964b. The genetical evolution of social behaviour. II. J Theor Biol 7(1):17–52. 5875340 10.1016/0022-5193(64)90039-6

[zoaf060-B26] Hammerschmidt K, Fischer J, 1998. Maternal discrimination of offspring vocalizations in Barbary macaques (*Macaca sylvanus*). Primates 39(2):231–236.

[zoaf060-B27] Hepper PG , 1986. Kin recognition: functions and mechanism. A review. Biol Rev Camb Philos Soc 61(1):63–93. 3521752 10.1111/j.1469-185x.1986.tb00427.x

[zoaf060-B28] Heth G, Todrank J, Johnson RE, 1998. Kin recognition in golden hamsters: evidence for phenotype matching. Anim Behav 56(2):409–417. 9787032 10.1006/anbe.1998.0747

[zoaf060-B29] Hothorn T, Bretz F, Westfall P, 2008. Simultaneous inference in general parametric models. Biom J 50(3):346–363. 18481363 10.1002/bimj.200810425

[zoaf060-B30] Janik VM, Knörnschild M, 2021. Vocal production learning in mammals revisited. Philos Trans R Soc Lond B Biol Sci 376(1836):20200244. 34482736 10.1098/rstb.2020.0244 PMC8419569

[zoaf060-B31] Janik VM, Slater PJB, 1998. Context specific use suggests that bottlenose dolphin signature whistles are cohesion calls. Anim Behav 56(4):829–835. 9790693 10.1006/anbe.1998.0881

[zoaf060-B32] Janus M , 1992. Interplay between various aspects in social relationships of young rhesus monkeys: dominance, agonistic help, and affiliation. Am J Primatol 26(4):291–308. 31948151 10.1002/ajp.1350260406

[zoaf060-B33] Kappeler PM , 2019. A framework for studying social complexity. Behav Ecol Sociobiol 73(1):13.

[zoaf060-B34] Keenan S, Mathevon N, Stevens JMG, Nicolè F, Zuberbühler K et al, 2020. The reliability of individual vocal signature varies across the bonobo's graded repertoire. Anim Behav 169:9–21.

[zoaf060-B35] Kessler SE, Radespiel U, Hasiniaina AIF, Nash LT, Zimmermann E, 2018. Does the grey mouse lemur use agonistic vocalisations to recognize kin? Contrib Zool 87(4):261–274.

[zoaf060-B36] King SL, Friedman WR, Allen SJ, Gerber L, Jensen FH et al, 2018. Bottlenose dolphins retain individual vocal labels in multi-level alliances. Curr Biol 28(12):1993–1999. 29887310 10.1016/j.cub.2018.05.013

[zoaf060-B37] Kirkpatrick RC, Grueter CC, 2010. Snub-nosed monkeys: multilevel societies across varied environments. Evol Anthropol 19(3):98–113.

[zoaf060-B38] Kondo N, Watanabe S, 2009. Contact calls: information and social function. Jpn Psychol Res 51(3):197–208.

[zoaf060-B39] Kreiman J, Lee Y, 2025. Biological, linguistic, and individual factors govern voice quality. J Acoust Soc Am 157(1):482–492. 39846773 10.1121/10.0034848

[zoaf060-B40] Langehennig-Peristenidou A, Scheumann M, 2024. Sex differences in the impact of social relationships on individual vocal signatures in grey mouse lemurs (*Microcebus murinus*). Philos Trans R Soc Lond B Biol Sci 379(1905):20230193. 38768201 10.1098/rstb.2023.0193 PMC11391318

[zoaf060-B41] Lemasson A, Hausberger M, 2004. Patterns of vocal sharing and social dynamics in a captive group of Campbell's monkeys (*Cercopithecus campbelli campbelli*). J Comp Psychol 118(3):347–359. 15482063 10.1037/0735-7036.118.3.347

[zoaf060-B42] Lemasson A, Hausberger M, 2011. Acoustic variability and social significance of calls in female Campbell's monkeys (*Cercopithecus campbelli campbelli*). J Acoust Soc Am 129(5):3341–3352. 21568434 10.1121/1.3569704

[zoaf060-B43] Lemasson A, Ouattara K, Petit EJ, Zuberbühler K, 2011. Social learning of vocal structure in a nonhuman primate? BMC Evol Biol 11(1):362. 22177339 10.1186/1471-2148-11-362 PMC3260242

[zoaf060-B44] Levréro F, Carrete-Vega G, Herbert A, Lawabi I, Courtiol A et al, 2015. Social shaping of voices does not impair phenotype matching of kinship in mandrills. Nat Commun 6(1):7609. 26139329 10.1038/ncomms8609

[zoaf060-B45] Levréro F, Touitou S, Frédet J, Nairaud B, Guéry JP et al, 2019. Social bonding drives vocal exchanges in bonobos. Sci Rep 9(1):711. 30679444 10.1038/s41598-018-36024-9 PMC6346008

[zoaf060-B46] Li Y, Guo S, Ji W, He G, Wang X et al, 2011. Social play behavior in infant Sichuan snub-nosed monkeys (*Rhinopithecus roxellana*) in Qinling Mountains, China. Am J Primatol 73(9):845–851. 21538447 10.1002/ajp.20944

[zoaf060-B47] Li B, Zhao D, 2007. Copulation behavior within one-male groups of wild *Rhinopithecus roxellana* in the Qinling Mountains of China. Primates 48(3):190–196. 17219093 10.1007/s10329-006-0029-7

[zoaf060-B48] Liu X, Stanford CB, Yang J, Yao H, Li Y, 2013. Foods eaten by the Sichuan snub-nosed monkey (*Rhinopithecus roxellana*) in Shennongjia National Nature Reserve, China, in relation to nutritional chemistry. Am J Primatol 75(8):860–871. 23589133 10.1002/ajp.22149

[zoaf060-B49] Marshall AJ, Wrangham RW, Arcadi AC, 1999. Does learning affect the structure of vocalizations in chimpanzees? Anim Behav 58(4):825–830. 10512656 10.1006/anbe.1999.1219

[zoaf060-B50] McComb K, Moss C, Sayiadel S, Baker L, 2000. Unusually extensive networks of vocal recognition in African elephants. Anim Behav 59(6):1103–1109. 10877888 10.1006/anbe.2000.1406

[zoaf060-B51] McComb K, Redy D, Baker L, Moss C, Sayiadel S, 2003. Long-distance communication of acoustic cues to social identity in African elephants. Anim Behav 65(2):317–329.

[zoaf060-B52] Meyer D, Hodges JK, Rinaldi D, Wijaya A, Roos C et al, 2012. Acoustic structure of male loud-calls supports molecular phylogeny of Sumatran and Javanese leaf monkeys (genus *Presbytis*). BMC Ecol Evol 12:16–27. 10.1186/1471-2148-12-16 PMC3295661 22305415

[zoaf060-B53] Mine JG, Wilke C, Zulberti C, Behjati M, Bosshard AB et al, 2024. Vocal-visual combinations in wild chimpanzees. Behav Ecol Sociobiol 78(10):108.

[zoaf060-B54] Painter MC, Gustison ML, Snyder-Mackler N, Johnson ET, le Roux A et al, 2024. Acoustic variation and group level convergence of gelada, *Theropithecus gelada*, contact calls. Anim Behav 207:235–246.

[zoaf060-B55] Pardo JS , 2006. On phonetic convergence during conversational interaction. J Acoust Soc Am 119(4):2382–2393. 16642851 10.1121/1.2178720

[zoaf060-B56] Penn D, Frommen J, 2010. Kin recognition: an overview of conceptual issues, mechanisms and evolutionary theory. In: Keppeler P, editor. Animal Behaviour: Evolution and Mechanisms. Berlin, Heidelberg: Springer, 55–85.

[zoaf060-B57] Pfefferle D, Hammerschmidt K, Mundry R, Ruiz LAV, Fischer J et al, 2016. Does the structure of female rhesus macaque coo calls reflect relatedness and/or familiarity. PLoS One 11(8):e0161133. 27579491 10.1371/journal.pone.0161133 PMC5007041

[zoaf060-B58] Pfefferle D, Kazem AJN, Brockhausen RR, Ruiz-Lambides AV, Widdig A, 2014. Monkeys spontaneously discriminate their unfamiliar paternal kin under natural conditions using facial cues. Curr Biol 24(15):1806–1810. 25065751 10.1016/j.cub.2014.06.058 PMC4137972

[zoaf060-B59] Pfefferle D, Ruiz-Lambides AV, Widdig A, 2013. Female rhesus macaques discriminate unfamiliar paternal sisters in playback experiments: support for acoustic phenotype matching. Proc Biol Sci 281(1774):20131628. 24225452 10.1098/rspb.2013.1628 PMC3843825

[zoaf060-B60] Pollard KA, Blumstein DT, 2011. Social group size predicts the evolution of individuality. Curr Biol 21(5):413–417. 21333537 10.1016/j.cub.2011.01.051

[zoaf060-B61] Poole JH, Tyack PL, Stoeger-Horwath AS, Watwood S, 2005. Animal behaviour: elephants are capable of vocal learning. Nature 434(7032):455–456. 15791244 10.1038/434455a

[zoaf060-B62] Pusey AE , 1987. Sex-biased dispersal and inbreeding avoidance in birds and mammals. Trends Ecol Evol 2(10):295–299. 21227869 10.1016/0169-5347(87)90081-4

[zoaf060-B63] Qi X, Grueter CC, Fang G, Huang Z, Zhang J et al, 2020. Multilevel societies facilitate infanticide avoidance through increased extrapair matings. Anim Behav 161:127–137.

[zoaf060-B64] Qi X, Huang K, Fang G, Grueter CC, Dunn DW et al, 2017. Male cooperation for breeding opportunities contributes to the evolution of multilevel societies. Proc Biol Sci 284(1863):20171480. 28954911 10.1098/rspb.2017.1480 PMC5627208

[zoaf060-B65] Qi X, Li B, Garber PA, Ji W, Watanabe K, 2009. Social dynamics of the golden snub-nosed monkey (*Rhinopithecus roxellana*): female transfer and one-male unit succession. Am J Primatol 71(8):670–679. 19434626 10.1002/ajp.20702

[zoaf060-B66] Qi X, Wu J, Zhao L, Wang L, Guang X et al, 2023. Adaptations to a cold climate promoted social evolution in Asian colobine primates. Science 380(6648):eabl8621. 37262163 10.1126/science.abl8621

[zoaf060-B67] R Core Team , 2024. R: A Language and Environment for Statistical Computing. Vienna, Austria: R Foundation for Statistical Computing.

[zoaf060-B68] Rebout N, Lone J, De Marco A, Cozzolino R, Lemasson A et al, 2021. Measuring complexity in organisms and organizations. R Soc Open Sci 8(3):200895. 33959307 10.1098/rsos.200895 PMC8074971

[zoaf060-B69] Ren Y, Huang K, Guo S, Pan R, Derek DW et al, 2018. Kinship promotes affiliative behaviors in a monkey. Curr Zool 64(4):441–447. 30108624 10.1093/cz/zox046 PMC6084570

[zoaf060-B70] Ren B, Zhang S, Xia S, Li Q, Liang B et al, 2003. Annual reproductive behavior of *Rhinopithecus roxellana*. Int J Primatol 24(3):575–589.

[zoaf060-B71] Riede T , 2010. Elasticity and stress relaxation of rhesus monkey (*Macaca mulatta*) vocal folds. J Exp Biol 213(17):2924–2932. 20709920 10.1242/jeb.044404 PMC2922750

[zoaf060-B72] Ruch H, Zürcher Y, Burkart JM, 2018. The function and mechanism of vocal accommodation in humans and other primates. Biol Rev Camb Philos Soc 93(2):996–1013. 29111610 10.1111/brv.12382

[zoaf060-B73] Rukstalis M, Fite JE, French JA, 2003. Social change affects vocal structure in a Callitrichid primate (*Callithrix kuhlii*). Ethology 109(4):327–340.

[zoaf060-B74] Silk JB, Altmann J, Alberts SC, 2006. Social relationships among adult female baboons (*Papio cynocephalus*). I. Variation in the strength of social bonds. Behav Ecol Sociobiol 61(2):183–195.

[zoaf060-B75] Slocombe KE, Zuberbühler K, 2006. Food-associated calls in chimpanzees: responses to food types or food preferences? Anim Behav 72(5):989–999.

[zoaf060-B76] Smith-Vidaurre G, Perez-Marrufo V, Wright TF, 2021. Individual vocal signatures show reduced complexity following invasion. Anim Behav 179:15–39.

[zoaf060-B77] Smith K, Alberts SC, Altmann J, 2003. Wild female baboons bias their social behaviour towards paternal half-sisters. Proc Biol Sci 270(1514):503–510. 12641905 10.1098/rspb.2002.2277 PMC1691261

[zoaf060-B78] Snowdon CT, Cleveland J, 1980. Individual recognition of contact calls by pygmy marmosets. Anim Behav 28(3):717–727.

[zoaf060-B79] Snowdon CT, Elowson AM, 1999. Pygmy marmosets modify call structure when paired. Ethology 105(10):893–908.

[zoaf060-B80] Snowdon CT, Hausberger M, 1997. Social Influences on Vocal Development. Cambridge: Cambridge University Press.

[zoaf060-B81] Soltis J, Leong K, Savage A, 2005. African elephant vocal communication II: rumble variation reflects the individual identity and emotional state of callers. Anim Behav 70(3):589–599.

[zoaf060-B82] Sugiura H , 2007. Effects of proximity and behavioral context on acoustic variation in the coo calls of Japanese macaques. Am J Primatol 69(12):1412–1424. 17508342 10.1002/ajp.20447

[zoaf060-B83] Sussman RW, Garber PA, 2011. Cooperation and competition in primate social interactions. In: Campbell CJ, Fuentes A, MacKinnon KC, Panger M, Bearder SK, editors. Primates in Perspective. New York: Oxford University Press, 587–599.

[zoaf060-B84] Tanaka T, Sugiura H, Masataka N, 2006. Cross-sectional and longitudinal studies of the development of group differences in acoustic features of coo calls in two groups of Japanese macaques. Ethology 112(1):7–21.

[zoaf060-B85] Tang-Martinez Z , 2001. The mechanisms of kin discrimination and the evolution of kin recognition in vertebrates: a critical re-evaluation. Behav Process 53(1-2):21–40. 10.1016/s0376-6357(00)00148-0 11254989

[zoaf060-B86] Thinh VN, Hallam C, Roos C, Hammerschmidt K, 2011. Concordance between vocal and genetic diversity in crested gibbons. BMC Evol Biol 11(1):36. 21299843 10.1186/1471-2148-11-36 PMC3044664

[zoaf060-B87] Tibbetts EA, Dale J, 2007. Individual recognition: it is good to be different. Trends Ecol Evol 22(10):529–537. 17904686 10.1016/j.tree.2007.09.001

[zoaf060-B88] Tyack PL , 2000. Dolphins whistle a signature tune. Science 289(5483):1310–1311. 10979857 10.1126/science.289.5483.1310

[zoaf060-B89] Tyack PL , 2008. Convergence of calls as animals form social bonds, active compensation for noisy communication channels, and the evolution of vocal learning in mammals. J Comp Psychol 122(3):319–331. 18729661 10.1037/a0013087

[zoaf060-B90] Wada K, Li B, Watanabe K, 2015. Affiliative interactions between one-male units in a band of Sichuan snub-nosed monkeys (*Rhinopithecus roxellana*) living in the Qinling Mountains, China. Primates 56(4):327–337. 26162774 10.1007/s10329-015-0475-1

[zoaf060-B91] Wang X, Wang C, Qi X, Guo S, Zhao H et al, 2013. A newly-found pattern of social relationships among adults within one-male units of golden snub-nosed monkeys (*Rhinopithecus roxellana*) in the Qinling Mountains, China. Integr Zool 8(4):400–409. 24344964 10.1111/1749-4877.12026

[zoaf060-B92] Weiss MN, Franks DW, Croft DP, Whitehead H, 2019. Measuring the complexity of social associations using mixture models. Behav Ecol Sociobiol 73(1):8.

[zoaf060-B93] Widdig A , 2007. Paternal kin discrimination: the evidence and likely mechanisms. Biol Rev Camb Philos Soc 82(2):319–334. 17437563 10.1111/j.1469-185X.2007.00011.x

[zoaf060-B94] Widdig A, Nürnberg P, Krawczak M, Streich WJ, Bercovitch FB, 2001. Paternal relatedness and age proximity regulate social relationships among adult female rhesus macaques. Proc Natl Acad Sci U S A 98(24):13769–13773. 11698652 10.1073/pnas.241210198 PMC61116

[zoaf060-B95] Xiang Z, Yang B, Yu Y, Yao H, Grueter CC et al, 2014. Males collectively defend their one-male units against bachelor males in a multi-level primate society. Am J Primatol 76(7):609–617. 24375453 10.1002/ajp.22254

[zoaf060-B96] Xiang Z, Yu Y, Yao H, Hu Q, Yang W et al, 2022. Female countertactics to male feticide and infanticide in a multilevel primate society. Behav Ecol 33(4):679–687.

[zoaf060-B97] Yang Y, Yan Y, Fang G, Song Y, Zang J et al, 2024. Functional diversification of contact calls contributes to the cohesion of a multi-level society. Behav Ecol Sociobiol 78(12):121.

[zoaf060-B98] Yao H, Liu X, Stanford CB, Yang J, Huang T et al, 2011. Male dispersal in a provisioned multilevel group of *Rhinopithecus roxellana* in Shennongjia Nature Reserve, China. Am J Primatol 73(12):1280–1288. 21898518 10.1002/ajp.21000

[zoaf060-B99] Zar JH , 1999. Biostatistical Analysis. Upper Saddle River (NJ): Prentice-Hall.

[zoaf060-B100] Zhang P, Li B, Qi X, Macintosh AJJ, Watanabe K, 2012. A proximity-based social network of a group of Sichuan snub-nosed monkeys (*Rhinopithecus roxellana*). Int J Primatol 33(5):1081–1095.

[zoaf060-B101] Zhang P, Watanabe K, Li B, 2008. Female social dynamics in a provisioned free-ranging band of the Sichuan snub-nosed monkey (*Rhinopithecus roxellana*) in the Qinling Mountains, China. Am J Primatol 70(11):1013–1022. 18615543 10.1002/ajp.20591

[zoaf060-B102] Zhao D, Li B, 2009. Do deposed adult male Sichuan snub-nosed monkeys *Rhinopithecus roxellana* roam as solitary bachelors or continue to interact with former band members? Curr Zool 55(3):235–237.

[zoaf060-B103] Zuberbühler K, Byrne RW, 2006. Social cognition. Curr Biol 16(18):R786–R790. 16979541 10.1016/j.cub.2006.08.046

[zoaf060-B104] Zürcher Y, Willems EP, Burkart JM, 2021. Trade-offs between vocal accommodation and individual recognisability in common marmoset vocalizations. Sci Rep 11(1):15683–15694. 34344939 10.1038/s41598-021-95101-8 PMC8333328

