# Supplementary material for: Acoustic analysis in relation to kinship and familiarity for contact calls of adult female golden snub-nosed monkeys living in a multilevel society
Source: Curr Zool. 2025 Aug 23;72(4):500–11. doi: 10.1093/cz/zoaf060 (PMC13500171; doi:10.1093/cz/zoaf060)
Supplement: Supplementary file 1 [file zoaf060_supplementary_data.docx]

**Table S1. Effects of kinship, length of time co-resident in the same social unit, and age difference, and their interaction terms on structural similarities of contact calls between individuals of adult female golden snub-nosed monkeys**

|  | Estimate | SE | t-value |
| --- | --- | --- | --- |
| (Intercept) | 1.555 | 0.131 | 11.886 |
| Distant kin | –0.030 | 0.152 | –0.198 |
| Close kin | –0.221 | 0.156 | –1.411 |
| Short time | –0.386 | 0.371 | –1.040 |
| Medium time | –0.049 | 0.503 | –0.097 |
| Long time | –0.355 | 0.239 | –1.486 |
| Age difference | 0.389 | 0.048 | 8.110 |
| Two-way interactions: |  |  |  |
| Distant kin * short time | –0.134 | 0.447 | –0.300 |
| Distant kin * medium time | 0.192 | 0.547 | 0.351 |
| Distant kin * long time | 0.495 | 0.367 | 1.347 |
| Distant kin * age difference | –0.026 | 0.064 | –0.412 |
| Close kin * short time | 0.297 | 0.525 | 0.566 |
| Close kin * medium time | –0.055 | 0.537 | –0.103 |
| Close kin * long time | 0.488 | 0.301 | 1.619 |
| Close kin * age difference | 0.068 | 0.065 | 1.035 |
| Short time * age difference | 0.064 | 0.182 | 0.352 |
| Medium time * age difference | –0.031 | 0.284 | –0.111 |
| Long time * age difference | 0.061 | 0.120 | 0.512 |
| Three-way interactions: |  |  |  |
| Distant kin * short time * age difference | 0.123 | 0.217 | 0.568 |
| Distant kin * medium time * age difference | –0.044 | 0.299 | –0.147 |
| Distant kin * long time * age difference | –0.075 | 0.175 | –0.428 |
| Close kin * short time * age difference | –0.102 | 0.265 | –0.384 |
| Close kin * medium time * age difference | 0.014 | 0.296 | 0.048 |
| Close kin * long time * age difference | –0.163 | 0.148 | –1.107 |

Log likelihood ratio test: all predictors and interaction terms (*χ*^2^ = 281.280, *df* = 23, *P* < 0.001), three-way interaction (*χ*^2^ = 2.894, *df* = 6, *P* = 0.822), kinship * age difference (*χ*^2^ = 2.625, *df* = 2, *P* = 0.269), length of time co-resident in the same unit * age difference (*χ*^2^ = 2.973, *df* = 3, *P* = 0.396), kinship * length of time co-resident in the same unit (*χ*^2^ = 6.441, *df* = 6, *P* = 0.376).

**Table S2. The standardized canonical coefficients and eigenvalues of, and variance explained by discriminant functions (DF) generated by a stepwise discriminant analysis for the individuality of contact calls from adult female golden snub-nosed monkeys**

| Parameter | DF_1_ | DF_2_ | DF_3_ | DF_4_ | DF_5_ | DF_6_ |
| --- | --- | --- | --- | --- | --- | --- |
| *f*_0__mean | 1.892 | 2.456 | –0.500 | 0.438 | 0.931 | 0.319 |
| *f*_0__range | 1.037 | 2.196 | –0.129 | 1.315 | 1.015 | 0.552 |
| *f*_0__max | –1.004 | –2.099 | 0.164 | –1.805 | –1.077 | –0.717 |
| *f*_0__Q_3_ | –0.994 | –1.042 | 0.415 | –0.161 | –0.097 | –0.383 |
| *f*_0__SD | –0.470 | –1.375 | 0.141 | 0.473 | –0.494 | 0.024 |
| *f*_0__min | 0.396 | –0.298 | 0.450 | 0.450 | –0.218 | 0.197 |
| *f*_0__IQR | 0.312 | 0.341 | –0.426 | –0.268 | 0.048 | 0.450 |
| A_IQR | –0.236 | 0.210 | –0.168 | 0.299 | 0.282 | 0.353 |
| *f*_0__Q_2_ | –0.221 | –0.336 | 0.235 | –0.269 | –0.103 | –0.125 |
| HNR | –0.210 | 0.092 | 0.235 | –0.554 | –0.390 | 0.066 |
| Shimmer | –0.207 | –0.043 | 0.121 | –0.439 | –0.199 | 0.187 |
| A_SD | 0.203 | –0.480 | –0.009 | –0.683 | –0.126 | –0.247 |
| A_Q_3_ | 0.191 | –0.061 | 0.496 | –0.187 | –0.071 | 0.225 |
| A_end | 0.180 | –0.142 | –0.184 | –0.254 | 0.023 | –0.009 |
| %T_max*f*_0_ | –0.162 | –0.063 | 0.043 | 0.259 | –0.132 | 0.000 |
| Jitter | 0.149 | –0.390 | –0.058 | 0.046 | 0.199 | 0.078 |
| Duration | –0.146 | 0.426 | 0.387 | 0.097 | 0.468 | –0.012 |
| %T_minA | 0.138 | –0.093 | –0.123 | –0.063 | –0.123 | –0.125 |
| A_Q_2_ | –0.130 | 0.182 | 0.369 | 0.018 | –0.173 | 0.160 |
| ΔF | 0.120 | –0.103 | 0.616 | 0.352 | –0.181 | –1.059 |
| *f*_0__start | 0.114 | 0.044 | –0.038 | –0.008 | 0.125 | –0.391 |
| A_mean | –0.080 | –0.464 | –0.995 | –0.603 | 0.637 | –0.468 |
| A_min | –0.065 | 0.807 | 0.848 | 1.904 | –0.103 | 0.279 |
| *f*_0__end | –0.049 | 0.093 | –0.113 | 0.401 | –0.308 | 0.241 |
| A_range | 0.040 | 1.029 | 0.594 | 1.829 | –0.227 | 0.814 |
| %T_min*f*_0_ | 0.040 | 0.257 | –0.173 | 0.083 | –0.101 | 0.065 |
| A_start | 0.035 | 0.187 | 0.027 | 0.170 | 0.253 | –0.132 |
| *f*_0__Q_1_ | 0.023 | –0.337 | 0.175 | –0.061 | –0.012 | 0.110 |
| F_2_ | –0.015 | 0.376 | –0.319 | 0.005 | –0.361 | –0.682 |
| F_3_ | –0.008 | –0.161 | –0.290 | 0.083 | –0.008 | 0.081 |
| %T_maxA | 0.006 | 0.184 | –0.097 | –0.109 | 0.124 | –0.071 |
| F_1_ | –0.004 | –0.521 | 1.276 | 0.258 | 0.192 | –0.627 |
| F_4_ | 0.004 | –0.008 | –0.609 | –0.139 | 0.490 | 0.866 |
|  |  |  |  |  |  |  |
| Eigenvalues | 6.246 | 0.881 | 0.756 | 0.557 | 0.437 | 0.392 |
| Variance explained | 53.8% | 7.6% | 6.5% | 4.8% | 3.8% | 3.4% |

**Figure S1. Structural similarities of contact calls between individuals of adult female golden snub-nosed monkeys. (a) Structural similarities were expressed as the F values generated by a stepwise discriminant analysis and were sorted from the smallest to the largest. (b) Normal Q-Q plot of logarithmic transformed F values.**
